# Class II HLA-DRB4 is a predictive biomarker for survival following immunotherapy in metastatic non-small cell lung cancer

**DOI:** 10.1038/s41598-023-48546-y

**Published:** 2024-01-03

**Authors:** Cindy Y. Jiang, Lili Zhao, Michael D. Green, Shashidhar Ravishankar, Andrea M. H. Towlerton, Anthony J. Scott, Malini Raghavan, Matthew F. Cusick, Edus H. Warren, Nithya Ramnath

**Affiliations:** 1 Department of Internal Medicine, University of Michigan, Ann Arbor, MI USA; 2 Division of Cancer Medicine, The University of Texas MD Anderson Cancer Center, Houston, TX USA; 3 Department of Biostatistics, University of Michigan, Ann Arbor, MI USA; 4 Department of Radiation Oncology, University of Michigan, Ann Arbor, MI USA; 5 Clinical Research Division, Fred Hutchinson Cancer Research Center, Seattle, WA USA; 6 Division of Clinical Genetics, Department of Internal Medicine, University of Michigan, Ann Arbor, MI USA; 7 Department of Microbiology and Immunology, University of Michigan, Ann Arbor, MI USA; 8 Department of Pathology, University of Michigan, Ann Arbor, MI USA; 9 Lieutenant Colonel Charles S. Kettles VA Medical Center (VA Ann Arbor Health System), 2215 Fuller Ave, Ann Arbor, MI 48105 USA; 10 Division of Hematology/Oncology, Department of Internal Medicine, University of Michigan, Ann Arbor, MI USA

**Keywords:** Lung cancer, Cancer, Cancer immunotherapy

## Abstract

Immune checkpoint inhibitors (ICI) are important treatment options for metastatic non-small cell lung cancer (mNSCLC). However, not all patients benefit from ICIs and can experience immune-related adverse events (irAEs). Limited understanding exists for germline determinants of ICI efficacy and toxicity, but Human Leukocyte Antigen (HLA) genes have emerged as a potential predictive biomarker. We performed HLA typing on 85 patients with mNSCLC, on ICI therapy and analyzed the impact of HLA Class II genotype on progression free survival (PFS), overall survival (OS), and irAEs. Most patients received pembrolizumab (83.5%). HLA-DRB4 genotype was seen in 34/85 (40%) and its presence correlated with improved OS in both univariate (*p* = 0.022; 26.3 months vs 10.2 months) and multivariate analysis (*p* = 0.011, HR 0.49, 95% CI [0.29, 0.85]). PFS did not reach significance (univariate, *p* = 0.12, 8.2 months vs 5.1 months). Eleven patients developed endocrine irAEs. HLA-DRB4 was the predominant genotype among these patients (9/11, 81.8%). Cumulative incidence of endocrine irAEs was higher in patients with HLA-DRB4 (*p* = 0.0139). Our study is the first to suggest that patients with metastatic NSCLC patients on ICI therapy with HLA-DRB4 genotype experience improved survival outcomes. Patients with HLA-DRB4 had the longest median OS (26.3 months). Additionally, we found a correlation between HLA-DRB4 and the occurrence of endocrine irAEs.

## Introduction

In the United States, lung cancer is the third most common cancer with over 236,700 new cases in 2022 and is the leading cause of cancer deaths, accounting for 24% of all cancer-related deaths^1^. Around 85% of lung cancer cases are non-small cell lung cancer (NSCLC)^2^. While surgery and stereotactic body radiotherapy are potentially curative for early stage disease, the majority of patients present with locally advanced or metastatic disease which is more refractory to treatment^3^. Historically, for patients with locally advanced disease, the five-year survival after chemoradiation was 32%^4^, and for patients with metastatic disease, the two-year survival after chemotherapy was 11%^5^.

Immune checkpoint inhibitors (ICIs) have considerably improved the outcomes of patients with all stages of lung cancer. The ICIs used in NSCLC target the programmed death 1 (PD-1) receptor, the programmed death ligand 1 (PD-L1), or cytotoxic T-lymphocyte-associated protein 4 (CTLA4). Neoadjuvant nivolumab (anti-PD-1) plus ipilimumab (anti-CTLA4) prior to surgical resection of Stage I/II NSCLC results in complete pathologic responses in as many as 38% of patients^6^. Resectable NSCLC patients who received neoadjuvant nivolumab in combination with chemotherapy experienced improved 24-month progression-free survival (PFS) of 77%^7^ and 24% of patients had a complete pathologic response^8^. The addition of adjuvant durvalumab, a PD-L1 inhibitor, following chemoradiation in Stage III NSCLC improves the 5-year overall survival to 43%^9^. Finally, the use of pembrolizumab (anti-PD-1) in metastatic NSCLC patients with tumor PD-L1 score > 50%, has improved the 5-year overall survival to 31%^10^. Other regimens include: (1) combination of chemotherapy (platinum-based plus pemetrexed) and pembrolizumab, (2) atezolizumab (anti-PD-L1)+/− chemotherapy and bevacizumab (VEGF inhibitor), (3) cemiplimab (anti-PD-1)+/− chemotherapy, (4) nivolumab plus ipilimumab+/– chemotherapy^11–15^. This has led to widespread use of ICI across all stages of NSCLC. However, not all patients experience benefit from ICIs and identification of robust predictive biomarkers remains an unmet need.

Currently, only three predictive biomarkers have been approved by the US FDA for ICI therapy in cancers, namely programmed death-ligand 1 (PD-L1), microsatellite instability (MSI) or defective mismatch repair (dMMR), and tumor mutational burden (TMB). Of these, tumor PD-L1 expression on tumor cells plays an important role in treatment decision making for NSCLC^16^. There has been exploration of other potential biomarkers, such as tumor infiltrating lymphocytes (TIL), specific gut microbiota, various microRNAs, and peripheral blood markers (i.e. neutrophil to lymphocyte ratio and lactate dehydrogenase), but few predictive biomarkers have been validated^16–18^. Human leukocyte antigens (HLA) are encoded by major histocompatibility complex (MHC) genes and have emerged as an area of strong interest as predictive biomarkers following ICI use^19^. HLA class I proteins (HLA A, B, and C) are widely expressed on all nucleated cells and present antigens to CD8+ T cells^20^. HLA class II proteins (HLA DP, DQ, DR) have limited expression predominantly on antigen presenting cells (i.e. dendritic cells, macrophages, B cells) and present antigens to CD4^+^ T cells^21^. The HLA system is critical for self versus non-self-discrimination by the immune system as well as for the detection of cancer. Loss of HLA Class I has emerged as an important immune evasion mechanism in NSCLC^22^ and specific HLA class I alleles have been associated with ICI efficacy and toxicity^23–27^. For example, HLA-A*03 and HLA-B62 supertype have been found to predict poor response to immunotherapy, while HLA-B*44 supertype predicts for improved survival^28,29^. In most studies, patients are more commonly tested for HLA class I genotypes. This has led to limited clinical studies examining the association between HLA class II antigens and ICI efficacy. Currently, available studies note that increased HLA class II related gene expression correlates with improved outcomes^30^. Notably, higher HLA-DR expression has been suggested to predict response to ICI^31,32^.

ICIs are associated with a unique spectrum of side effects known as immune-related adverse events (irAEs). irAEs can affect any organ, with the most commonly affected organs including the skin, colon, liver, lungs, and endocrine glands^33^. While the majority of irAEs in patients with NSCLC are low grade, severe side effects requiring therapy discontinuation occur in up to 20% of Stage III NSCLC patients^34^ and 17% of patients with Stage IV NSCLC^35^. Fulminant and fatal toxicities may occur^36^. Management relies on immunosuppression with glucocorticoids, tumor necrosis factor alpha antagonists, or other agents, but, on occasion, these therapies have resulted in tumor progression^37^. Immune checkpoints play a role in limiting autoimmune disease, thus use of checkpoint inhibitors may trigger autoimmune inflammation in normal tissues. Interestingly, it has been noted that irAEs occur with higher frequency in patients with pre-existing autoimmune antibodies^38^. However, there is limited understanding of whether germline determinants predispose to irAE development.

Herein, we examined patients with metastatic NSCLC who received ICIs and performed HLA typing in an agnostic manner for all classical HLA loci (HLA-A, -B, -C, -DRB1, -DRB3/4/5, -DQA1, -DQB1, -DPA1, and -DPB1) and discovered an overrepresentation of the class II alleles, specifically DRB4 among patients with endocrine irAEs. We also found that carriers of HLA-DRB4 had a longer overall survival. Herein, we present data from our observations on HLA genotypes in patients with metastatic NSCLC treated with ICI and specifically present correlations between Class II HLA-DRB4 and ICI efficacy and irAE.

## Methods

### Patients

The data on HLA genotypes was gathered from a larger prospective study that involved the collection of baseline tumor biopsies and blood samples in patients being treated with ICIs for metastatic NSCLC to identify biomarkers predictive of therapeutic response and toxicity. The study was conducted at the Veterans Administration, Ann Arbor Healthcare System (VAAAHS). Enrollment for the study began in November 2015 and ended in June 2022. Study eligibility for patients included diagnosis of metastatic NSCLC and initiation of ICI therapy. A total of 85 patients with clinical outcomes and complete HLA genotype data are available. All patients were reviewed for the development of any adverse event and both classification and grading were based on the Common Terminology Criteria for Adverse Events version 5.0 (CTCAE). Response assessment was performed using Response Evaluation Criteria in Solid Tumors Version 1.1** (**RECIST V1.1). Our study did not include an additional review of adverse events by independent investigators.

### HLA genotyping data

HLA genotyping was completed via ScisGo HLA typing kits (Scisco Genetics, Seattle, WA). The full standardized protocol is available online^39^. DNA was extracted from patient blood and this was followed by genomic DNA quantification and library preparation using the ScisGo HLA Typing Kit. HLA genotypes of each patient were directly compared to each other and closely analyzed for similarities and the presence of HLA loci known to be associated with autoimmune endocrine disorders^40,41^.

### Statistical analysis

Descriptive statistics of the clinical and demographic data included mean, median, and range for numerical variables as well as percentages for categorical variables. Important clinical and demographic data included gender, race, histology, smoking history, performance status, and prior therapies. Progression free survival (PFS) was defined as time from ICI start to radiographic disease progression, defined by RECIST V1.1. Overall survival (OS) was defined as time from ICI start to death or date of last follow up. Kaplan–Meier method was used to estimate the PFS and OS functions, and log-rank test was used for the comparisons. Cox proportional hazards regression was used to assess the association between HLA-DRB4 and PFS/OS, adjusting for age, gender, race, stage, smoking history, and histology. To further study the association of irAE to survival, we included it as a time-varying covariate in the Cox model. The time-varying covariate had a value of 0 before the irAE and 1 after irAE; for patients who did not have irAE, this covariate had a value of 0 during the entire follow-up period. SAS (version 9.4) was used for the analyses and significance was defined by a two-tailed p-value < 0.05. To assess the association between HLA-DRB4 and the development of endocrine irAEs, cumulative incidence functions were used and significance was based on Gray’s test.

### Study approval

This clinical study was approved by the VA Ann Arbor Healthcare System institutional review board and ethics committee. Written informed consent was obtained from all patients. All research was performed in accordance with relevant guidelines and regulations, including the Declaration of Helsinki.

### Ethics approval

Approved by VA Ann Arbor Healthcare System institutional review board and ethics committee.

### Consent to participate

Informed consent was obtained from all individual participants included in the study.

## Results

### Patient demographics

There were 98 eligible cancer patients with metastatic NSCLC enrolled in this study. Of these, 85 (86%) had complete clinical data and adequate baseline DNA for HLA genotype analysis. Most patients were men (96.5%), the majority were former/active smokers (98.8%), and the median age was 72 years (IQR 66–75). The patients were predominantly Caucasian (75.3%). Before receiving ICI therapy, 21.2% had received chemotherapy only and 49.4% had received both chemotherapy and radiation therapy. All patients who received chemotherapy were exposed to platinum regimens (n = 60). The average number of prior therapies (prior to immunotherapy) was 1 (range 0–2). Other therapies tried before immunotherapy included carboplatin/pemetrexed, cisplatin/etoposide, carboplatin/paclitaxel, carboplatin/etoposide, carboplatin/gemcitabine, cisplatin/vinorelbine, cisplatin/gemcitabine, carboplatin/pemetrexed/bevacizumab, and cisplatin/docetaxel. Most patients received pembrolizumab (83.5%) with 14 receiving pembrolizumab in conjunction with carboplatin and pemetrexed. Other ICIs included durvalumab and nivolumab. 20 patients (23.5%) developed irAEs: 11 with endocrine irAEs (diabetes = 1; thyroiditis = 5, adrenal insufficiency = 2, and both = 3) and 9 with other irAEs (Table 1).

**Table 1:** Patient demographics and clinical characteristics.

	Pts w/o Endocrine irAEs (n = 74)	Pts w/Endocrine irAEs (n = 11)
Age, years—median (IQR)	69 (65.3–73.8)	70 (65–72.5)
Sex		
Male—No. (%)	71 (95.9)	11 (100)
Female—No. (%)	3 (3.5)	0 (0)
Self-identified race		
White, Caucasian—No. (%)	55 (74.3)	9 (81.8)
African American—No. (%)	9 (12.2)	1 (9.1)
Hawaiian or Pacific Islander—No. (%)	2 (2.7)	0 (0)
Did not declare—No. (%)	8 (10.8)	1 (9.1)
Histology		
Squamous—No. (%)	26 (35.1)	5 (45.5)
Adenocarcinoma—No. (%)	43 (58.1)	5 (45.5)
Adenosquamous—No. (%)	0 (0)	1 (9.1)
Poorly differentiated—No. (%)	4 (5.4)	0 (0)
Unknown—No. (%)	1 (1.4)	0 (0)
Charleston comorbidity index—mean (range)	9.7 (4–13)	9.8 (9–14)
Pack years—Mean (range)	50.6 (1–165)	43.0 (0–110)
Former smoker—No. (%)	48 (64.9)	8 (72.7)
Active smoker—No. (%)	26 (35.1)	2 (18.2)
Never smoker—No. (%)	0 (0)	1 (9.1)
Prior therapies		
Prior chemotherapy only—No. (%)	14 (18.9)	4 (36.4)
Prior radiation therapy only—No. (%)	14 (18.9)	0 (0)
Prior chemotherapy + radiation—No. (%)	37 (50.0)	7 (63.6)
No prior therapy—No. (%)	9 (12.2)	0 (0)
ICI therapy		
Pembrolizumab—No. (%)	62 (83.8)	10 (90.9)
Nivolumab—No. (%)	4 (5.4)	0 (0)
Durvalumab—No. (%)	8 (10.8)	1 (9.1)
Number of ICI cycles—median (range)	5 (1–53)	8 (3–35)
Durable clinical benefit		
Yes—No. (%)	30 (40.5)	9 (81.8)
No—No. (%)	42 (56.8)	2 (18.2)
Unknown—No. (%)	2 (2.7)	0 (0)
Immune related adverse event		
Thyroiditis only—No. (%)	0 (0)	5 (45.5)
AI only—No. (%)	0 (0)	2 (18.2)
Both thyroiditis and AI—No. (%)	0 (0)	3 (27.3)
Diabetes—No. (%)	0 (0)	1 (9.1)
Encephalitis—No. (%)	1 (1.4)	0 (0)
Arthralgia—No. (%)	1 (1.4)	0 (0)
Pneumonitis—No. (%)	2 (2.7)	0 (0)
Bullous pemphigoid—No. (%)	1 (1.4)	0 (0)
Pruritis—No. (%)	1 (1.4)	0 (0)
Rash—No. (%)	2 (2.7)	0 (0)
Transaminitis—No. (%)	1 (1.4)	0 (0)
Time from ICI initiation to irAE, months—median (range)	4.6 (2.8–27.5)	5.37 (1.5–58.6)
Status at last follow-up		
Deceased—No. (%)	55 (74.3)	5 (45.5)
Alive—No. (%)	19 (25.7)	6 (54.5)
Discontinued ICI therapy—No. (%)	64 (86.5)	11 (100)

### Overall treatment efficacy

The median follow-up time for all 85 patients was 42.8 months (IQR 23.7–63.6). The median PFS was 6.7 months (IQR 2.1–20.9) and median OS was 13.2 months (IQR 6.05–33.9). 39/85 (45.9%) patients experienced durable clinical benefit, which was defined as a response or stable disease on therapy at ≥ 6 months. 9 patients remained alive for ≥ 36 months.

### HLA characteristics

We assessed the HLA genotype in all patients (see Supplementary Fig. 1 heat map). Overall, the most common HLA class I types were HLA-A*02 (n = 43), HLA-A*03 (n = 27), and HLA-C*07 (n = 46). The most frequent HLA class II genotypes were HLA-DPA1*01 (n = 82), HLA-DPB1*04 (n = 62), and HLA-DQA1*01 (n = 61). Functional HLA-DRB4*01 was carried in 34 patients. Of note, 4 patients carried HLA-DRB4*01:03:01:02N (null allele) without a second functional HLA-DRB4 allele and 1 patient expressed HLA-DRB4*01:03:02, which was classified as a null allele. Patients carrying only null alleles were not included in the total for patients carrying HLA-DRB4*01. However, patients carrying HLA*DRB4*01:03:01:02N along with a second functional HLA-DRB4*01:03:01 allele were included in the analysis (n = 3). There were 25 patients carrying HLA-DRB4 and HLA-DRB1*04, 15 patients carrying HLA-DRB4 and HLA-DRB1*07, and 2 patients carrying HLA-DRB4 and HLA-DRB1*09.

### HLA-DRB4 allele variation

Allelic variation is an important determinant of HLA function^42^. To explore whether specific HLA-DRB4 subtypes were associated with ICI treatment tolerance, we examined these more closely. In total, 28 patients carried HLA-DRB4*01:03:01. Six patients carried HLA-DRB4*01:01:01. All the patients with both thyroiditis and adrenal insufficiency (3/3) carried HLA-DRB4*01:03:01 as well as 40% of the patients with thyroiditis only (2/5) and one patient with diabetes (1/1). Two patients with adrenal insufficiency and one patient with thyroiditis carried the HLA-DRB4*01:01:01 allele. HLA-DRB4 *01:03:01 was carried in 44.4% (4/9) of patients who developed other types of irAEs. In patients who did not develop any irAEs, HLA-DRB4 was carried in 32% (n = 21), with HLA-DRB4*01:03:01 allele predominating in 86% of the patients (n = 18), HLA-DRB4*01:01:01 in 11.5% of the patients (n = 3). Two out of the 34 patients carrying HLA-DRB4 were homozygous with one of these patients developing endocrine irAEs. The remaining patients did not carry HLA-DRB4 as the second allele (n = 32).

### Correlation between HLA genotype and survival

**Figure 1:**
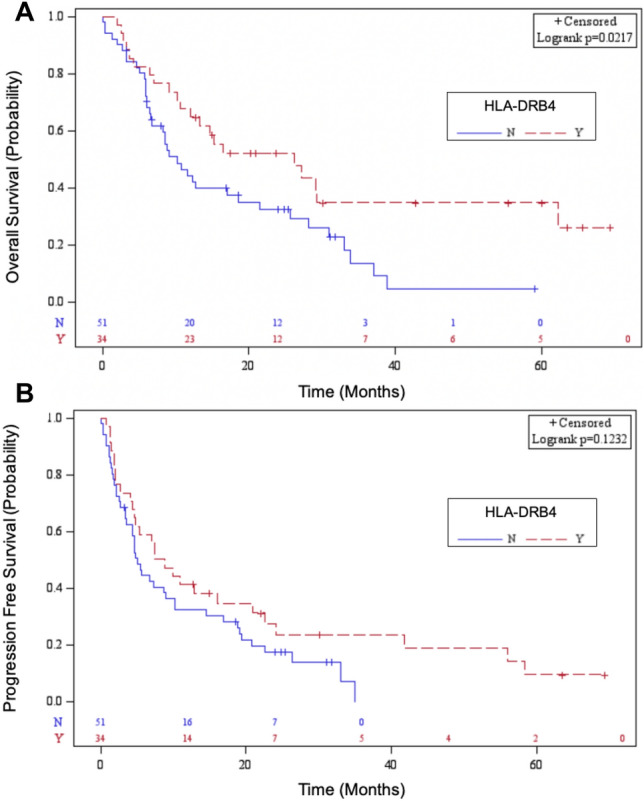
Kaplan Meier Analysis of Survival in Patients Stratified by HLA-DRB4 Status (**A**) Improved overall survival in patients carrying HLA-DRB4. (**B**) No change in progression free survival with HLA-DRB4. N = HLA-DRB4 not carried, Y = HLA-DRB4 carried.

Patients who carried HLA-DRB4 had improved OS at 26.3 months in comparison to 10.2 months in patients who did not carry HLA-DRB4 (*p* = 0.022) (Fig. 1A). However, PFS was not statistically different (*p* = 0.12; 8.2 months vs 5.1 months) (Fig. 1B). Comparison of specific alleles HLA-DRB4*01:03:01 versus HLA-DRB4*01:01:01 did not reveal any significant correlation with PFS (*p* = 0.64) *or* OS (*p* = 0.27). Of note, we also assessed the impact of HLA-A*03 on survival. There were 27 patients (31.8%) with HLA-A*03. HLA-A*03 was associated with a mOS of 10.7 months in comparison to 15.3 months in patients without HLA-A*03 (*p* = 0.74, 95% CI [6.5, 25.6]) and mPFS of 4.8 months versus 7.5 months (*p* = 0.99, 95% CI [2.8, 9.0]). Additional results of other HLA types and survival are available in Supplementary Table 1.

### Multivariable analysis

After adjusting for age, gender, race, stage, and histology, HLA-DRB4 was associated with improved OS (*p* = 0.011, HR 0.49, 95% CI [0.29, 0.85]) and approached significance for PFS (*p* = 0.051, HR 0.61, 95% CI [0.37, 1.00]).

### Description of irAEs

Of the 85 patients identified, 11 developed endocrine irAEs (12.9%). Ten (90.9%) patients received pembrolizumab and 1 received durvalumab (9.1%). The most frequent toxicity grade was 1 (n = 5). Overall, patient clinical characteristics and demographics were similar between those who developed endocrine irAEs and those who did not (Table 1). Thyroiditis was the most common endocrine irAE (n = 5) followed by both thyroiditis and adrenal insufficiency (n = 3), adrenal insufficiency alone (n = 2), and diabetes (n = 1). No patients in this cohort experienced new onset hyperthyroidism or hypoparathyroidism. The median time from treatment start to development of endocrine irAE was 5.4 months (IQR 2.87). The management and impact of endocrine irAEs on patients is outlined in Table 2.

**Table 2:** Clinical description of patients with endocrine irAEs.

Age	70	72	68	71	64	65	70	78	62	73	65
Gender	Male	Male	Male	Male	Male	Male	Male	Male	Male	Male	Male
Ethnicity*	White	White	White	White	White	White	Unknown	White	AA	White	White
ICI Therapy	Pembro	Pembro	Pembro	Pembro	Pembro	Pembro	Pembro	Pembro	Pembro	Pembro	Durva
Prior Endo Conditions	Yes—hypothyroid 2/2 Radiation	Yes—Hypothyroid	No	No	No	No	No	No	No	No	No
Endocrine irAE**	T	T; AI	T	AI	T	T; AI	T; AI	T	DM	AI	T
irAE Grade	1	1	2	3	2	3	1; 2	1	2	3	1
Time to Onset—months	1.47	4.13; 9.27	3.73	14.93	4.20	3.27; 3.73	5.37; 10.0	5.60	6.6	58.6	6.4
Other irAEs	No	Colitis	No	No	No	No	No	No	No	Colitis, dermatitis	Pneumonitis
Initial Treatment***	L	L; H then P, back to H	L; P	P	L; P	L; P	L; H	L	Metformin, lantus, aspart	Steroid taper	L
Continued Treatment***	L	L; weaned off steroids	P	P	L	L	L; H	L	Metformin, lantus, aspart	H	L
TSH Baseline/ After ICI	5.29/11.69	4.40/7.17	0.9/116	0.45/2.75	0.72/76.3	2.79/97.6	3.37/8.57	2.64/21.7	0.80/1.03	5.18/7.54	2.66/5.26
AM Cortisol	13.7	1.4	–	1.7	2.6	10.7	0.7	7.6	< 0.1	13	18.6
ACTH	–	10	–	< 5	–	22	5	–	< 0.1	–	< 0.1
Cosyntropin Simulation Test	–	7.1 → 11.6– > 14.3	–	1.0– > 0.7– > 9.6	–	–	–	–	–	–	–
ICI Status post-irAE	Continued until PD	Continued until surveillance	Held 2/2 irAE	Held 2/2 irAE	Held 2/2 irAE	Discontinued prior to irAE given PD	Continued until PD	Held 2/2 irAE	Held 2/2 irAE	Held 2/2 irAE	Held 2/2 irAE

*Ethnicity: AA = African American; **Endocrine irAE: T = thyroiditis, AI = adrenal insufficiency, DM = diabetes mellitus; ***Initial Treatment/Continued Treatment: L = levothyroxine, H = hydrocortisone, P = prednisone.

### Endocrine irAEs and HLA characteristics

**Figure 2:**
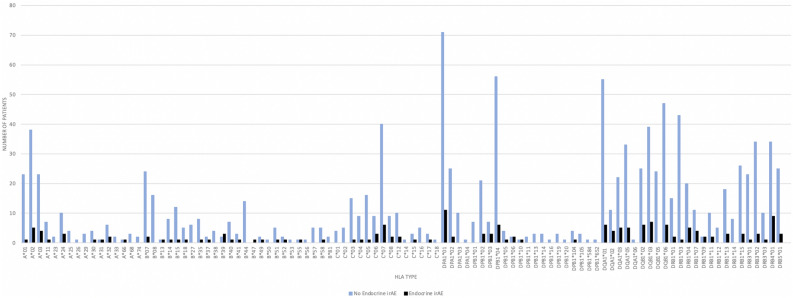
Comparison of HLA class I and II genotypes between Patients with and without Endocrine irAEs.

Among the 11 patients that developed endocrine irAEs, we noted HLA-C*07 (n = 7) and HLA-A*02 (n = 5) as the most predominant Class I HLA gene and HLA-DPA1*01 (n = 11) and HLA-DRB4*01 (n = 9) as the most frequent Class II HLA genes (Fig. 2). The Allele Frequency Net Database notes that HLA-DPA1*01 is very common in the United States (US) Amerindian population and the European Caucasians with 95% of individuals having the allele^43^. In comparison, HLA-DRB4 is less common in the US Caucasian population and present in approximately 40–50% of individuals, this was estimated based on limited available data^43^. Thirty-four (40%) patients in our cohort carried HLA-DRB4, with 9/11 (81.8%) patients with endocrine irAEs carrying the HLA-DRB4 genotype (Table 3). Given known co-expression of HLA-DRB4 and DRB1 alleles, we looked for this association, among patients with irAEs. There were 6 patients carrying both HLA-DRB4 and HLA-DRB1*04, 3 patients with both HLA-DRB4 and HLA-DRB1*07, and no patients carrying both HLA-DRB4 and HLA-DRB1*09.

**Table 3:** Correlation of HLA-DRB4 presence in Patients with and without Endocrine irAEs and Other irAEs. Significant values are in bold.

	HLA-DRB4 present	HLA-DRB4 not present	Total
Pts w/endocrine irAEs (%)	9 (81.8)	2 (18.2)	**11**
Pts w/other irAEs (%)	4 (44.4)	5 (55.6)	**9**
Pts w/o irAEs (%)	21 (32.3)	44 (67.7)	**65**
Total	**34**	**51**	**85**

### Association between HLA-DRB4 and irAEs

**Figure 3:**
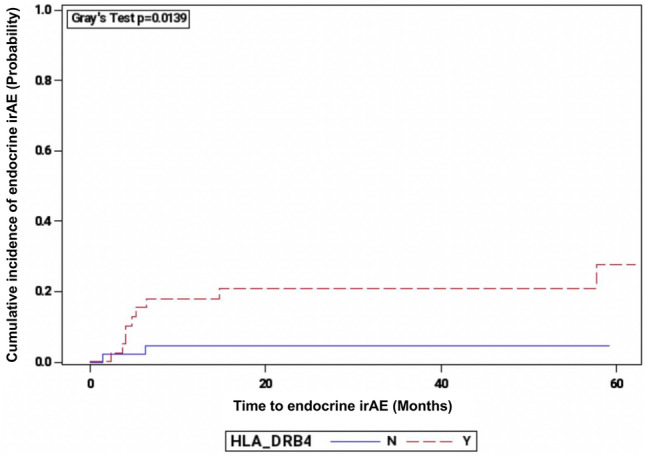
Cumulative Incidence of Endocrine irAEs Stratifid by HLA-DRB4 Status. N = HLA-DRB4 not carried, Y = HLA-DRB4 carried.

Patients were assessed for the cumulative incidence of endocrine irAEs over time based on HLA-DRB4. We found that patients who carry HLA-DRB4 were statistically more likely to develop endocrine irAEs compared to those who did not carry HLA-DRB4 (p = 0.0139 by Gray’s test, Fig. 3). We also assessed the cumulative incidence of endocrine irAEs over time based on HLA-A*03 and there was no significant association (p = 0.68 by Gray’s test). Patients carrying both HLA-DPA1*01 and HLA-DRB4 did not have an increased likelihood of developing endocrine irAEs (*p* = 0.20* by Gray’s test).* Similarly, carrying both HLA-DRB4 and DRB1*04 did not correlate with increased incidence of endocrine irAEs (*p* = 0.60* by Gray’s test*) nor did carrying both HLA-DRB4 and DRB1*07 (*p* = 0.36 by Gray’s test). Additional results of the cumulative incidence of endocrine irAEs and other HLA types are available in Supplementary Table 2.

### Correlation between irAEs and survival

To investigate if development of irAEs correlated with ICI therapy efficacy, we next stratified patients by development of toxicity and evaluated survival outcomes. The presence of any irAE (endocrine or other) did not statistically improve PFS (*p* = 0.226, HR 0.61, 95% CI [0.268, 1.365]) or OS *(p* = 0.219, HR 0.63, 95% CI [0.301,1.316]). When comparing survival outcomes between presence of endocrine and other irAEs, there was again no statistically significant improvement in PFS (*p* = 0.530, HR 0.5, 95% CI [0.230–17.4]).

### Multivariable analysis

After adjusting for age, gender, race, stage, and histology, the development of any irAE did not improve PFS (*p* = 0.085), HR 0.339, 95% CI [0.099, 1.162]) or OS (*p* = 0.275, HR 0.636, 95% CI [0.282, 1.434]). Multivariable analysis of endocrine irAE patients was not possible given the small sample size.

## Discussion

Immunotherapy with ICI has significantly improved lung cancer outcomes. However, many patients derive limited benefit from ICI therapy and some patients experience toxicities ranging from mild to fulminant. There are many reasons for lack of benefit to ICIs. Preclinical and translational studies have identified primary and acquired mechanisms of resistance^44^ including hepatic siphoning^45^, tumoral loss of HLA Class I^46^, T cell chemokine silencing^47^, and immunometabolic checkpoints^48^. In parallel, preclinical and translational studies have begun to identify potential cellular mediators of irAEs, including hepatitis^49^, thyroiditis^50^, and colitis^51^. Given the importance of antigen presentation to immune responses, studies are beginning to link HLA genotypes to ICI efficacy and toxicity. However, many of these studies have focused on HLA class I genotypes rather than HLA class II genotypes.

Our study revealed a significant correlation between HLA-DRB4 and improved OS, on both univariable and multivariable analysis. To our knowledge, this is the first such report. Patients carrying HLA-DRB4 had the longest median OS of 26.3 months. We also explored the impact of DRB4 allelic variation on survival but did not find any significant differences between the allelic variations. It is important to note that our study was not powered to make definitive conclusions for allelic variations. Other studies have reported associations between MHC class II and ICI efficacy. Correale et al.^25^ noted longer survival in patients who were heterozygous for HLA DRB1, and Yang et al.^30^ noted increased expression of MHC class II was associated with improved survival. In their study, HLA-DMB, HLA-DOA, HLA-DPB1, and HLA-DMA were included in the top HLA genes related to outcomes. More recently, HLA-A*03 has been associated with inferior outcomes in patients receiving ICIs^28^. In our study, we did note a trend towards HLA-A*03 having worse survival outcomes, but it was not statistically significant. The lack of significance may be due to the small sample size of this study leading to inadequate power to detect a difference. Other groups have described an inverse correlation between loss of heterozygosity (LOH) for HLA Class I and II loci in tumors with survival. Schaafsma et al.^52^ reported that any presence of Class I or II is protective and may result in improved activity from immune checkpoint inhibitors. Interestingly, presence of any Class I or Class II in the tumor bearing cells was associated with improved survival in this cohort of patients, derived from multiple datasets of patients on ICIs. Importantly, this study was based on somatic expression and not germline expression. In NSCLC, HLA LOH occurs in 40% of early-stage cancers and is enriched in metastatic tumors^22^. Advanced cancer patients that were heterozygous at all HLA class I loci had improved survival as compared to patients who were homozygous at any one locus^29^. Furthermore, Schaafsma et al.^52^ observed significant increase in an HLA class II gene expression when comparing patients with and without clinical benefit in on-treatment samples as compared to pre-treatment samples, suggesting that on-treatment samples are more informative of clinical benefit when using HLA class II gene expression as an indicator of response. It is proposed that the activation of CD4+ T cells by HLA class II expression helps to initiate CD8+ T cells that consequently mount a successful antitumor immune response during ICI therapy^53^. Collectively, these data highlight the contribution of MHC class II mediated immune responses to therapeutic anti-tumor immune responses.

Secondarily, we found a significant association between HLA-DRB4 and incidence of endocrine irAEs. We found an overrepresentation of HLA-DRB4 in thirteen of the twenty patients with any degree of irAEs and nine of the eleven patients with endocrine irAEs. In comparison, only 40–50% of the general Caucasian population carry HLA-DRB4^43^. This observation has not been previously reported. Extensive genetic polymorphisms and high a degree of homology within the HLA locus make HLA typing challenging^43,54^. The exact mechanisms leading to the development of irAEs are not clear, however there have been translational studies have implicated autoreactive T cells, autoantibodies, and pro-inflammatory cytokines^55^. The blockade of checkpoints like PD-1 allows T-cells to react against self-antigens presented by HLA, and this in turn can led to inflammatory damage to normal organ tissue where these checkpoints are normally found^56^. HLA-DRB4 has been linked to a number of autoimmune diseases, including autoimmune hepatitis^57^, type 1 diabetes^58,59^, autoimmune myocarditis^60^, anti-LG1 encephalitis^61^, rheumatoid arthritis^62^, and juvenile idiopathic arthritis^63^. Notably, there are multiple reports of a significant association between HLA-DRB4 and development of Hashimoto’s Thyroiditis^64–66^. This finding demonstrates potential similarities between irAEs and autoimmune diseases, suggesting that certain HLA alleles may predispose to endocrine irAEs. Ongoing challenges with identifying a specific allele associated with development of disease include the MHC region having the highest gene density in the human genome, extended haplotypes having other plausible candidate genes, and associated diseases not presenting in a Mendelian inheritance pattern. Further investigation with family studies, a larger cohort, and more diverse population are necessary to explore these challenges. Additionally, future studies should assess the impact of linkage disequilibrium with stratification of HLA haplotypes.

Both retrospective and prospective studies have noted an association between development of irAEs and response to ICI therapy^67^. In one study describing patients with NSCLC treated with nivolumab, it was found that the median OS for patients who developed irAEs was significantly higher compared to those who did not develop irAE^68^. These findings have been confirmed in post hoc analyses from clinical trials as well as in prospective cohort studies^69^. Unlike in previous studies^70^, we did not find an association between development of endocrine irAEs and survival. Notably, patients in our study developed endocrine irAEs later in their treatment course with a median time to onset of 5.4 months. Overall, these studies highlight the significant linkage between immunotherapy efficacy and toxicity.

This study has several important limitations. While prospective, the study was not powered to detect survival differences between HLA-DRB4 allele subtypes and was not able to evaluate LOH in the HLA-DRB4 locus. Secondly, this is a single institution study with predominantly Caucasian male patients. Multi-institutional studies with more diverse patient populations and a larger cohort of patients will be needed. On treatment biopsies were not available, limiting insights into the cellular and molecular mechanisms through which HLA-DRB4 may contribute to immunotherapy efficacy and toxicity. Finally, the toxicity rate was overall relatively low, possibly secondary to predominately single agent ICI utilization.

In conclusion, our study is the first to report that HLA-DRB4 is associated with improved overall survival in metastatic NSCLC patients receiving ICI. We also found that HLA-DRB4 was associated with an increased likelihood of developing endocrine irAEs in patients receiving ICI. However, our study was small and future larger studies are needed to validate our findings of HLA-DRB4 as a predictive marker for survival and development of endocrine irAEs. Additionally, mechanistic studies aimed at understanding if there are conserved antigens presented by HLA-DRB4 that contribute to both irAE and anti-tumor immunity are needed.

### Supplementary Information

Supplementary Information.

## Data Availability

The dataset analyzed during this current study is available from the corresponding author upon request.
