# Supplementary material for: Class II HLA-DRB4 is a predictive biomarker for survival following immunotherapy in metastatic non-small cell lung cancer
Source: Sci Rep. 2024 Jan 3;14:345. doi: 10.1038/s41598-023-48546-y (PMC10764770; doi:10.1038/s41598-023-48546-y)
Supplement: Supplementary file 1 — Supplementary Information. [file 41598_2023_48546_MOESM1_ESM.docx]

|  | Median OS, months (p-value) | Median PFS, months (p-value) |
| --- | --- | --- |
| YES HLA-DPA1*01 vs NO HLA-DPA1*01 | 13 vs 12.3 (*p = 0.75)* | 6.7 vs 5.1 (*p = 0.94)* |
| YES HLA-DPA1*01 + HLA-DRB4 vs NO HLA-DPA1*01 + HLA-DRB4 | 26.2 vs 8.8 (***p = 0.009)*** | 9.9 vs 4.6 (***p = 0.04)*** |
| HLA-DRB4 + HLA-DRB1*04 vs HLA-DRB4 + HLA-DRB1*07 | 16.5 vs 27.2 *(p =0.29)* | 6.2 vs 16.7 (*p = 0.21)* |

**Supplementary Table 1. Correlation between Other HLA Types and Survival**

|  | p-value (by Gray’s Test) |
| --- | --- |
| HLA-A*03 | 0.68 |
| HLA-DPA1*01 + HLA-DRB4 | 0.20 |
| HLA-DRB4 + HLA- DRB1*04 | 0.60 |
| HLA-DRB4 + HLA- DRB1*07 | 0.36 |

**Supplementary Table 2. Incidence of Endocrine Immune Related Adverse Events and other HLA Types**


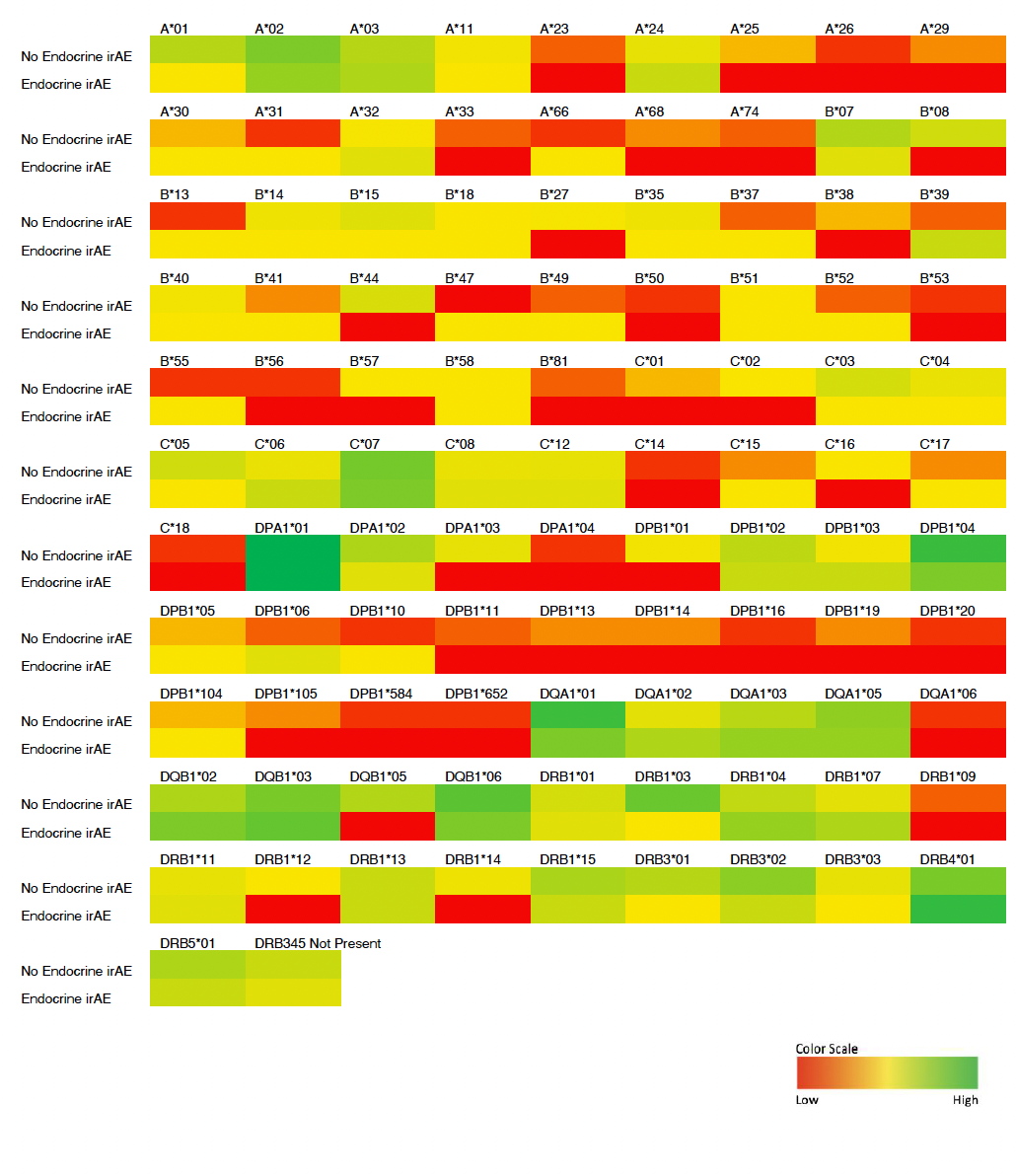


**Supplementary Figure 1. Heat Map Comparing HLA Type I and II Genotypes Between Patients with and without Endocrine Immune Related Adverse Events (irAEs)**
